# DARTS: an open-source Python pipeline for Ca^2+^ microdomain analysis in live cell imaging data

**DOI:** 10.3389/fimmu.2023.1299435

**Published:** 2024-01-11

**Authors:** Lena-Marie Woelk, Dejan Kovacevic, Hümeyra Husseini, Fritz Förster, Fynn Gerlach, Franziska Möckl, Marcus Altfeld, Andreas H. Guse, Björn-Philipp Diercks, René Werner

**Affiliations:** ^1^ Department of Applied Medical Informatics, University Medical Center Hamburg-Eppendorf, Hamburg, Germany; ^2^ Department of Computational Neuroscience, University Medical Center Hamburg-Eppendorf, Hamburg, Germany; ^3^ Center for Biomedical Artificial Intelligence (bAIome), University Medical Center Hamburg-Eppendorf, Hamburg, Germany; ^4^ The Calcium Signalling Group, Department of Biochemistry and Molecular Cell Biology, University Medical Center Hamburg-Eppendorf, Hamburg, Germany; ^5^ Institute for Immunology, University Medical Center Hamburg-Eppendorf, Hamburg, Germany

**Keywords:** intracellular signaling, Ca^2+^ microdomains, live cell imaging, image analysis, shape normalization, Python, open source

## Abstract

Ca^2+^ microdomains play a key role in intracellular signaling processes. For instance, they mediate the activation of T cells and, thus, the initial adaptive immune system. They are, however, also of utmost importance for activation of other cells, and a detailed understanding of the dynamics of these spatially localized Ca^2+^ signals is crucial for a better understanding of the underlying signaling processes. A typical approach to analyze Ca^2+^ microdomain dynamics is live cell fluorescence microscopy imaging. Experiments usually involve imaging a larger number of cells of different groups (for instance, wild type and knockout cells), followed by a time consuming image and data analysis. With DARTS, we present a modular Python pipeline for efficient Ca^2+^ microdomain analysis in live cell imaging data. DARTS (Deconvolution, Analysis, Registration, Tracking, and Shape normalization) provides state-of-the-art image postprocessing options like deep learning-based cell detection and tracking, spatio-temporal image deconvolution, and bleaching correction. An integrated automated Ca^2+^ microdomain detection offers direct access to global statistics like the number of microdomains for cell groups, corresponding signal intensity levels, and the temporal evolution of the measures. With a focus on bead stimulation experiments, DARTS provides a so-called dartboard projection analysis and visualization approach. A dartboard projection covers spatio-temporal normalization of the bead contact areas and cell shape normalization onto a circular template that enables aggregation of the spatiotemporal information of the microdomain detection results for the individual cells of the cell groups of interest. The dartboard visualization allows intuitive interpretation of the spatio-temporal microdomain dynamics at the group level. The application of DARTS is illustrated by three use cases in the context of the formation of initial Ca^2+^ microdomains after cell stimulation. DARTS is provided as an open-source solution and will be continuously extended upon the feedback of the community.

**Code available at:** 10.5281/zenodo.10459243.

## 1 Introduction

The activation of T cells, and thus the initial adaptive immune response, is mediated by an increase of the free cytosolic Ca^2+^ concentration. These signals are highly dynamic and spatially localized in the form of Ca^2+^ microdomains (1). There have been numerous studies investigating the molecular mechanisms and the role of Ca^2+^ signaling in early T cell activation in primary murine T cells (2–6) as well as Jurkat cells (2, 7). The studies show that the second messenger nicotinic acid adenine dinucleotide phosphate (NAADP), its binding protein named hematological and neurological expressed 1–like protein (HN1L) [also known as Jupiter microtubule-associated homolog 2 (JPT2)], and the type 1 ryanodine receptor (RyR1) are involved in the formation of subplasmalemmal Ca^2+^ signals that occur in the first seconds after T cell stimulation (7). Another essential component of the innate immune system are natural killer (NK) cells that contribute to the control of intracellular pathogens and cancer cells through direct cytotoxicity and the release of cytokines. To name a recent example, the NK cell status correlates with a decline in viral load in COVID-19 and thus can control SARS-CoV-2 replication by recognizing infected target cells (8). It has been shown that educated human NK cells have increased global Ca^2+^ signals compared to uneducated human NK cells following antibody stimulation (9). Similar to T cells, it is assumed that localized Ca^2+^ signals also arise after NK cell activation, but these Ca^2+^ microdomains have not been visualized so far. In line, in both cell types (and not only them), many questions about the molecular mechanisms behind the activation and signaling response are unsolved, and a better understanding of Ca^2+^ microdomain formation and dispersion is indispensable.

Fluorescence microscopy, and in particular live cell imaging, is an invaluable tool to study Ca^2+^ microdomains. As the relevant time frame is in the very early seconds after stimulation, high spatial and temporal resolution is crucial to obtain meaningful results. Even state-of-the-art imaging setups, however, encounter severe biological and physical constraints. Phototoxicity and photobleaching demand low photon doses, which, combined with out-of-focus light, lead to an intrinsically low signal-to-noise ratio (SNR). In addition, there is a trade-off between exposure times and imaging speed. Computational restoration of the acquired images, that is, a reduction of the noise level and correction for out-of-focus light, therefore has the potential to increase the SNR and to improve the analysis of Ca^2+^ microdomains in high-resolution live cell imaging data (10–13).

However, even when using computational image restoration approaches and deconvolution techniques, a key challenge remains for the comprehensive analysis of Ca^2+^ microdomains in live cell imaging data: It requires consistent and reliable, ideally automated, localization and quantification of Ca^2+^ microdomains across large ensembles of cells that often exhibit significant morphological changes between different cells and even within the same cell over time.

To address this challenge, in the present work, the modular pipeline DARTS (Deconvolution, Analysis, Registration, Tracking, and Shape normalization) for Ca^2+^ microdomain analysis in live cell fluorescence microscopy imaging data is introduced. The pipeline tackles imaging-related issues by providing problem-tailored image postprocessing routines (for instance, denoising and deconvolution, bleaching correction). Adapting a shape normalization technique introduced in (14), DARTS is able to compensate for morphological cell shape changes typically observed in the given application context. Moreover, DARTS offers automated detection of the microdomains in the live cell imaging data, the aggregation of the analysis results across cell ensembles, and intuitive dartboard visualization of the aggregated analysis results.

The individual DARTS parts and modules are explained in the following Method section. The Results section illustrates the use of DARTS with three application examples:

- The first use case illustrates the application of DARTS to the analysis of Ca^2+^ microdomains after stimulation of primary murine T cells, focusing on the comparison of wild type (WT) and RyR1 and RyR3 knockout (KO) cells (3).
- The second use case is based on a set of Jurkat T cells, in which the NAADP-binding protein HN1L/JPT2 is genetically deleted, resulting in a reduced formation of Ca^2+^ microdomains upon activation (7).
- As the third use case, DARTS is applied to the analysis of an NK cell line (KHYG-1) to illustrate that Ca^2+^ microdomains indeed arise after the activation of NK cells.

## 2 Methods

DARTS is implemented using an object-oriented approach in Python 3 and follows the open-source idea. The code as well as the software are provided under the most flexible license model (Apache 2.0 license). DARTS was tested for different operating systems (Windows, MacOS, Linux). The source code and a corresponding documentation can be found at github.com/ipmi-icns-uke/DARTS and ipmi-icns-uke.github.io/DARTS.

DARTS is intended both as a complete solution that can automatically process and analyze large volumes of cellular data “overnight” and as a toolkit from which individual modules can be selected, applied, and modified as needed. The intended and expected users are experimentalists with little or no programming experience. To support users without programming experience, a graphical user interface (GUI) is developed to help with parameter input. DARTS supports all common image input and output formats by integration of the Bio-Formats library (15).

**Figure 1:**
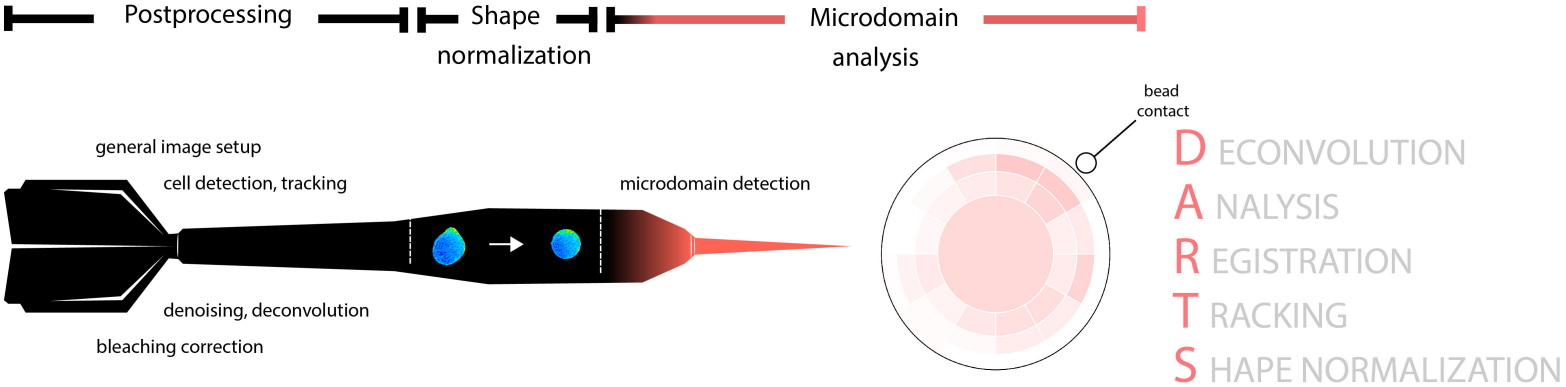
Schematic representation of the DARTS pipeline and its modules. The pipeline consists of three main parts: general image postprocessing; cell shape normalization; and Ca^2+^ microdomain detection, aggregation and visualization.

The general DARTS pipeline is illustrated in **Figure 1** and can be roughly divided into three main parts: live cell microscopy imaging data postprocessing; cell shape normalization; and Ca^2+^ microdomain detection and visualization.

### 2.1 DARTS image postprocessing modules

DARTS offers four image postprocessing modules: general image setup; cell detection and tracking; bleaching correction; and image denoising/deconvolution.

#### 2.1.1 General image setup: channel registration, ratio image computation, background subtraction

*Input:* temporal image sequence(s)

*Output:* temporal image sequence(s), same size as input sequences

Ratiometric imaging is a standard technique in fluorescence microscopy for imaging intracellular calcium signals. Depending on the imaging setup, the images obtained for the two calcium indicators are provided as imaging data of two often imperfectly aligned channels. As a default first processing step for ratiometric imaging, DARTS improves the alignment by an intensity-based affine registration of the images of the two channels, making use of the SimpleElastix library of the SimpleITK image analysis toolkit (16). Depending on the user’s preference, only the channel images of the first frame are registered and the resulting transformation is applied to all frames of the acquired time series data (time efficient solution, default) or all frames of the time series are individually registered and aligned (accurate alignment). Based on the aligned channel data, the desired ratio image is calculated (details vary, depending on the used calcium indicators) and, if desired, lower and upper thresholds are introduced to restrict the ratio image intensity values to the ratio value range of interest. Moreover, background subtraction can be applied to compensate for background fluorescence.

#### 2.1.2 Cell detection and tracking

*Input:* temporal image sequence, usually containing multiple cells

*Output:* temporal image sequence(s), each representing a region-of-interest containing a centered single cell

To allow analysis of Ca^2+^ microdomains at the single cell level and speed up subsequent analysis, DARTS performs automated detection and tracking of cells in the input live cell imaging sequence. The cells are identified in the individual frames of the input using the StarDist algorithm (17), a state-of-the-art algorithm that utilizes deep learning techniques to detect and segment cells in microscopy data. The result is a cell instance segmentation of the input image series at the frame level (cell representation: binary map, polyline of cell boundary). The detected individual cells are further tracked across time using the trackpy toolkit (18) in order to correctly position the regions of interest (ROI) and only retain the frames in which the cell is visible. For each cell that is successfully tracked (visible in a pre-defined number of frames, no large positional changes between consecutive frames), a new image time series is generated representing a rectangular region of interest (ROI) with the cell centered within the ROI, thus compensating translational movements of the cell.

#### 2.1.3 Bleaching correction

*Input:* temporal image sequence

*Output:* temporal image sequence, same size as image sequence

Calcium indicators often exhibit a relevant decrease of fluorescence intensity over time, commonly referred to as bleaching. DARTS provides an additive and a multiplicative bleaching correction that results in a frame-by-frame normalization of the mean cell pixel intensity to a constant value, and a biexponential fit-based additive correction (19). For ratiometric imaging, bleaching correction can be applied separately to the individual channel data.

#### 2.1.4 Image denoising/deconvolution

*Input:* temporal image sequence

*Output:* temporal image sequence, same size as image sequence

For reduction of out-of-focus light and image noise, two algorithms are provided: the standard Lucy-Richardson (LR) algorithm (20, 21) and the in-house developed time-dependent entropy (TDE) deconvolution (12). TDE can also be used as a re-implementation of the entropy-based image restoration approach by Arigovindan et al. (22), which specifically addresses the restoration of fluorescence signals in microscopy images with low signal-to-noise ratio (SNR). TDE extends the idea to time-series data, employing temporal consistency of signals over time as opposed to temporally independent noise patterns, and is particularly suited for live cell imaging data with low SNR, for instance due to short exposure times and high temporal resolution. For image data with reasonable SNR, however, the faster and parameter-free (excepting specification of the PSF) LR algorithm might be the more efficient choice.

### 2.2 DARTS shape normalization

*Input:* temporal image sequence, representing a ROI containing a centered single-cell

*Output:* temporal image sequence, representing a ROI containing the shape-normalized cell

Ca^2+^ microdomain analysis at a population level requires a spatially consistent evaluation of intracellular Ca^2+^ dynamics at the level of the individual cells of the population (for example, a consistent analysis of only the assembly of localized Ca^2+^ signals close to the contact point of a cell-activating bead). This is, depending on the variation in shape between different cells and also the degree of cell distortion over time, not always an easy task, because the relative position of intracellular signals, for instance with regard to the plasma membrane or the bead contact point, cannot always be directly described in a similar way for all cells and video frames. DARTS follows an approach introduced in (11): to map the individual cells to a common coordinate system and template of circular shape, respectively. For nearly circular cells like primary murine T cells, this step involves only a translation and scaling step. For other cells, the required transformation is more complex. DARTS adapts and modifies a shape normalization approach presented in (14). For each frame of the input image sequence (assumed to represent a ROI that contains a single cell to be analyzed), the coordinate system is converted from Cartesian to polar coordinates, (x, y) → (r, θ), where r is the radius and θ the polar angle. The centroid of the cell serves as the origin of the polar coordinate system. The coordinates of the cell border (r_o_, θ)_i=1,…,N_, for instance, computed by the DARTS cell detection and tracking module, are then mapped onto a circle with a radius determined by the mean radius value of the outline r_av_ = ∑_i=1,…,N_ r_o,i_/N, scaled by a constant factor. This corresponds to a coordinate transformation (r_o_, θ) → (r_o_’, θ) = T(r_o_, θ), with T being the transformation. To apply this transformation to the entire space of the considered ROI image, the transformation is interpolated using the nearest neighbor method and the inverse T^-1^(r', θ) = (r, θ) is used to obtain the pixel intensity values of the transformed image J(r', θ) from the corresponding locations in the source image I(r, θ).

### 2.3 DARTS Ca^2+^ microdomain analysis and dartboard projection

*Input:* temporal image sequences, each representing a ROI containing a centered single-cell

*Output:* analysis results at the single cell and the cell population level (text or Excel format)

The Ca^2+^ microdomain module workflow consists of two parts: frame-by-frame identification of the microdomains at the single-cell level and aggregation of the results for a cell population using a so-called dartboard projection.

Ca^2+^ microdomains are spatially localized areas of high Ca^2+^ concentration inside a cell. In DARTS, they are defined at a frame level and as small, connected sets of cell pixels with relative intensity values (normalized by the mean intensity of the cell pixels of the frame) above a predefined threshold. Both an appropriate definition of the size of pixel sets as well as the intensity threshold depends on the imaging setup (spatial and temporal microscopy resolution) and the applied postprocessing routines (for instance, deconvolution methods and additional image filters). It has to be carefully selected by the user, ideally combined with a comprehensive noise analysis of the setup. DARTS default values are chosen in agreement with (11). The single-cell microdomain analysis results (for instance the localization of the microdomains in each frame) can be exported as tables for further external signal analysis.

The dartboard projection analysis aims to aggregate and visualize the single-cell Ca^2+^ microdomain analysis results, in particular for the scenario of cell activation with a stimulatory bead. First, for each cell, the bead contact time and position relative to the center of the cell on a clock scale (1-12) needs to be defined by the user. DARTS provides a GUI for this step, where position and time point for each bead contact can be selected. To facilitate batch processing of larger data sets, the bead contact selection process is launched for all selected files before further postprocessing starts; all remaining processing steps are then fully automated and can be run “overnight”. Alternatively to the GUI-based bead contact selection, user input can be completely removed by providing a text file with the required information, for example generated by a previous run of the DARTS bead contact selection. To analyze and compare the microdomain locations and their appearance times relative to the contact within the different cells, the shape-normalized cells (output of the shape normalization module) are rotated such that the bead contact points of the cells are spatially aligned and temporally shifted such that the bead contacts are also temporally aligned. The structure of the dartboard is illustrated in **Figure 1**. The middle of the dartboard is defined as the frame-specific centroid coordinates of a shape-normalized cell image, and the outer border of the dartboard is aligned with the cell border. The identified Ca^2+^ microdomains are assigned to the different dartboard areas, depending on their angle relative to the bead contact position and the relative distance to the board center. The granularity of the dartboard analysis, that is, the number of dartboard rings and segments as well as the length of time periods used to aggregate the signals can be selected by the user, depending on the intended analysis scenario and cell lines. The radii of the rings of the dartboard decrease from inner to outer to ensure intuitive interpretation of the final results, that is, Ca^2+^ microdomain frequency within cell areas of identical size. To ensure comparability of all dartboard segments, results within a segment are normalized with respect to the individual segment area, thus compensating for the larger dartboard center (“bullseye”).

In principle, an averaging of the mere pixel intensities across the different cells of the considered cell set would also be possible after shape normalization. However, this would result in an unintended bias in the case of a global Ca^2+^ signal increase. In addition, the aggregation of local Ca^2+^ signals within larger spatial areas (the dartboard segments) allows a quick overview of the spatio-temporal distribution of Ca^2+^ microdomains even for medium-sized cell sets and an easy visual comparison of different data sets.

## 3 Results

The following application examples demonstrate the application of DARTS for three different cell types and lines.

### 3.1 Role of RYR1 and RYR3 in activation of primary murine T cells

The first application example corresponded to the Ca^2+^ microdomain analysis described in (3). It was based on primary murine WT and knockout CD4^+^ T cells. From the biological perspective, the imaging experiments were performed to investigate the role of type 1 ryanodine receptors 1 and 3 (RYR1, RYR3) in the formation of initial Ca^2+^ microdomains upon T cell stimulation. From a technical point of view, the application and imaging data were selected to illustrate the validity of the proposed DARTS pipeline in terms of reproducing the published observations and effects, despite the completely new implementation of the analysis parts compared to (11). In particular, primary murine T cells have a circular shape in the imaging data. Therefore, this example focused on the general workflow and not on specifics and potential problems encountered during shape normalization.

#### 3.1.1 Data set description

Primary murine CD4^+^ WT T cells as well as *Ryr1^-/-^* (2) and *Ryr3^-/-^* (23) on C57BL/6 J background (*Mus musculus*) were freshly isolated by negative selection using the EasySep Mouse CD4^+^ T Cell Enrichment Kit (STEMCELL Technologies Inc.). Image data were acquired by ratiometric Ca^2+^ imaging. Therefore, the freshly isolated T cells were loaded with Fluo4-AM (10 µM) and Fura Red-AM (20 µM) for 50 min at room temperature. The T cells were stimulated with protein G beads (Merck Millipore), coated with antibodies against CD3 and CD28 to mimic an immune synapse. Imaging was carried out with a Leica IRBE2 microscope using 100-fold magnification, a Sutter DG-4 as a light source and an electron-multiplying charge-coupled device (EMCCD) camera (Hamamatsu). Temporal resolution was 25 ms (40 frames/s) in 14-bit mode with twofold binning resulting in a spatial resolution of 368 nm. The emission wavelengths were split with a Dual-View module (Optical Insights, PerkinElmer Inc.) and the following filters were applied for the acquisition of initial Ca^2+^ microdomains (excitation (ex), 480/40; beam splitter (bs), 495; emission 1 (em1) 1, 542/50; em2, 650/57).

#### 3.1.2 Illustration of DARTS application

Since the data was acquired within a ratiometric imaging setup, all DARTS postprocessing modules were applied: channel registration, background subtraction, cell detection and tracking, deconvolution, bleaching correction, and ratio computation. Deconvolution was performed separately for the two-channel data using LR deconvolution, to be consistent with the procedure in (3). Since bleaching of Fluo4 was negligible, the bleaching correction was only applied to the Fura Red-channel data. Being interested in only Ca^2+^ microdomains, an additive correction was applied that normalizes the mean Fura Red intensity inside the cells for each frame to a constant value.

**Figure 2:**
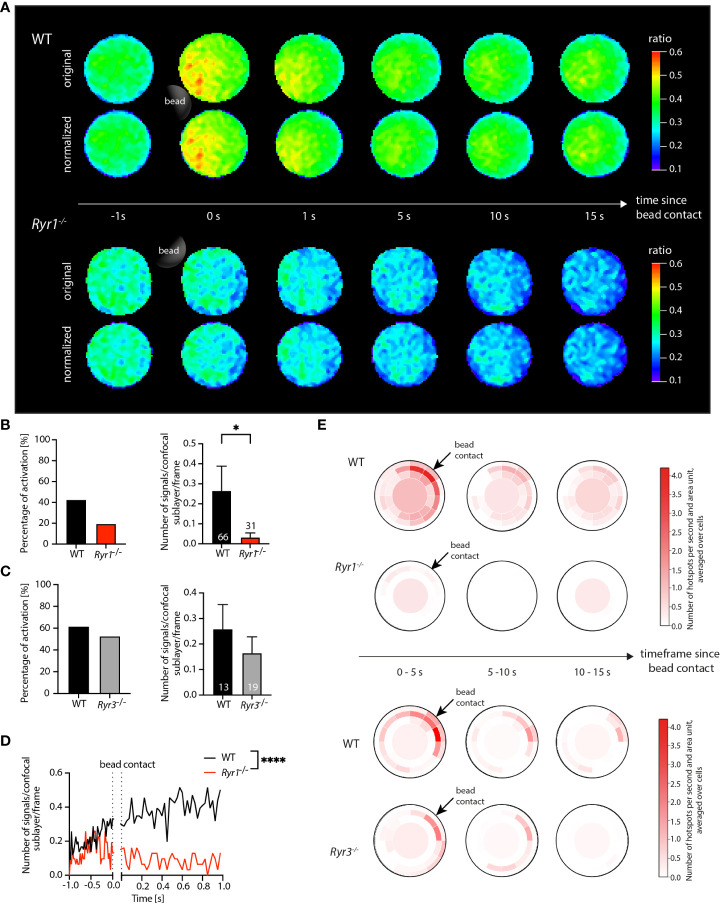
Application of DARTS to Ca^2+^ microdomain analysis in primary WT and *Ryr1* ^-/-^ and *Ryr3* ^-/-^ murine T cells. **(A)** Ca^2+^ signals in an exemplary WT T cell (top) and a *Ryr1* ^-/-^ T cell (bottom) upon the first seconds after bead contact. For both cells, the upper row shows the original and the lower row the shape-normalized cell representation. **(B, C)** Percentage of activated cells and number of Ca^2+^ microdomains within the first 15 s after bead stimulation; comparison for WT and *Ryr1^-/-^* and WT and *Ryr3^-/-^*, respectively. Data are presented as means ± SEM (WT, n = 66 cells, *Ryr1^-/-^*, n = 31 cells; WT, n = 13 cells, *Ryr3^-/-^*, n = 19 cells; statistical testing by Mann-Whitney-U-test, *p<0.05). **(D)** Number of Ca^2+^ microdomains as a function of time for WT (averaged across n = 66 cells) and *Ryr1^-/-^* cells (n = 31 cells; two-tailed Mann-Whitney-U-test, ****p<0.0001. **(E)** Dartboard projection plots of the WT cells in comparison to *Ryr1* ^-/-^ as well as WT in comparison to *Ryr3^-/-^*, showing the number of microdomains per second and dartboard segment area unit, averaged over the intervals 0-5 s, 5-10 s, and 10-15 s after bead contact and the same cells used for the global analysis shown in **(B, C)**. Bead contact location and time were aligned for all cells.

Following the application of the postprocessing modules, the ratio image series underwent the implemented shape normalization. Since primary T cells inherently exhibit a relatively circular shape, the shape-normalized ratio images closely resembled the original input ratio images and the original signal distribution (**Figure 2A**).

The results of the subsequent Ca^2+^ microdomain analysis are summarized in **Figures 2B–E**. The threshold for the detection of Ca^2+^ microdomains was based on a comprehensive noise analysis (3). Focusing on microdomain formation after the bead contact, analysis was performed for all frames from 1 second before and up to 15 seconds after bead contact.

The evaluation of the detected microdomains already allowed computation of global statistics such as the fraction of activated cells (cells that show microdomains from the bead contact on) and the number of signals per frame for each cell (**Figures 2B, C**). This information can be exported from DARTS to Excel or text format for subsequent detailed analysis in external programs. From the biological perspective, the global statistics revealed a significant difference between the number of microdomains per frame for the WT and the *Ryr1^-/-^* cells after bead contact (**Figure 2B**). The corresponding evaluation, but time-resolved and only for the period from 1 second before to 1 second after bead contact, is shown in **Figure 2D**. In contrast, no significant differences of the averaged number of signals between WT and *Ryr3^-/-^* cells for the present dataset was detected. The findings are consistent with the results in (3), illustrating that DARTS was able to reproduce the previous analysis results. Since it is a completely new and extended open-source implementation of the previous proprietary Matlab analysis workflow, perfect quantitative agreement is not expected. The new workflow now includes additional analysis steps like shape normalization to allow application to other use cases.

The dartboard projections are shown in **Figure 2E**; related dartboard information can also be exported from DARTS for subsequent analysis or visualization in external programs. Here, three dartboards for five-second intervals (0-5 s, 5-10 s, 10-15 s after bead contact) were generated. Representing the average number of hotspots per area unit and second, the dartboards exhibited a clear visual difference between WT and *Ryr1^-/-^* cells in the three time intervals. The *Ryr1^-/-^* cells barely showed any signals, especially close to the bead contact site, while the Ca^2+^ microdomain density in the WT cells was elevated in particular close to and directly after bead contact. In contrast, the patterns for WT and *Ryr3^-/-^* were similar, with a relatively high microdomain density close to the bead contact site, demonstrating that the detected Ca^2+^ signals that were summarized in the diagram in **Figure 2C** were indeed related to the bead stimulation of the cells. This information, not shown in (3), illustrates the additional value of spatially and temporally local analysis and information provided by the dartboard projections.

### 3.2 Human Jurkat cells: WT *vs*. *Hn1l*/*Jpt2* knockout

Continuing with the formation of initial Ca^2+^ microdomains in T cells, it has been reported that RYR1 is activated by nicotinic acid adenine dinucleotide phosphate (NAADP) (24). In (7), hematological and neurological expressed 1–like protein (HN1L), also known as Jupiter microtubule-associated homolog 2 (JPT2), was identified as a NAADP-binding protein. The conclusions drawn in (7) were (at least partly) based on initial Ca^2+^ microdomain analysis in Jurkat T cells. Compared to the primary murine T cells in section 3.1, Jurkat T cells exhibited considerable shape variation and deformation over time. Therefore, a direct application of a dartboard projection was not possible, and only global Ca^2+^ microdomains statistics (for instance, average number of signals for the entire cells) for WT and *Hn1l*/*Jpt2^-/-^* cells were presented in (7). Now, the DARTS workflow was applied to corresponding human Jurkat T cells to demonstrate that the shape normalization integrated in DARTS enables local spatiotemporal Ca^2+^ microdomain analysis even for cells with more complex shape and shape changes over time.

#### 3.2.1 Data set description

WT Jurkat T cells and *Hn1l/Jpt2^-/^* ^-^ Jurkat T cells (clone C2) were loaded with Fluo4-AM (10 µM) and Fura Red-AM (20 µM) for ratiometric single-cell Ca^2+^ imaging. The cells were stimulated with protein G beads (Merck Millipore) coated with antibodies against the T cell receptor CD3 (OKT-3) to mimic an immune synapse. Acquisition set-up for initial Ca^2+^ microdomains in Jurkat T cells was identical as described in section 3.1.

#### 3.2.2 Illustration of DARTS application

**Figure 3:**
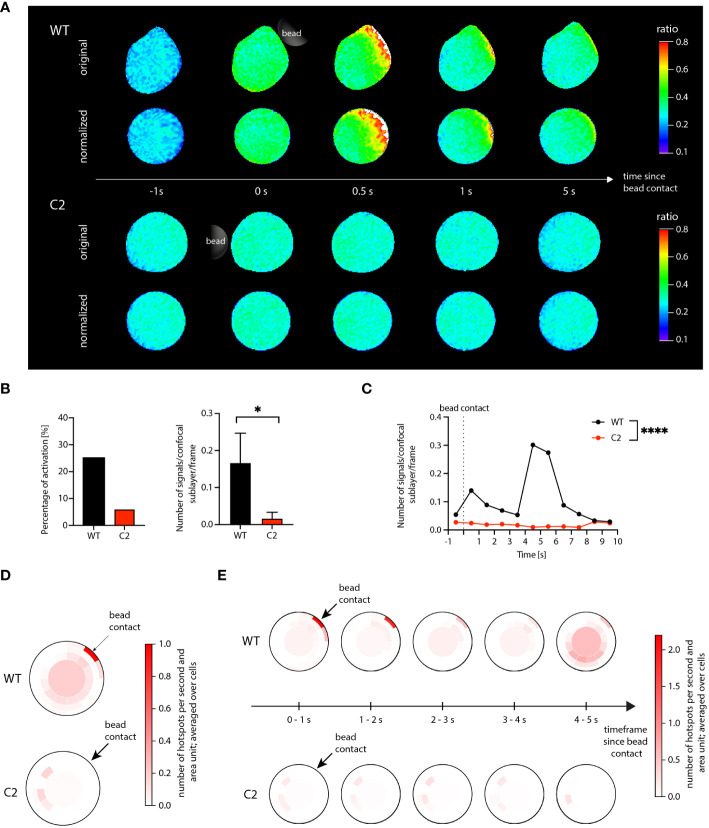
Ca^2+^ microdomain and dartboard analysis in Jurkat T cells. **(A)** Ca^2+^ signals in a WT Jurkat cell (top) and a *Hn1l/Jpt2^-/-^* (C2 clone) Jurkat cell (bottom) within the first seconds after stimulatory bead contact. For both cells, the upper row shows the original and the lower row the shape-normalized cell representation. **(B)** Global statistical analysis: percentage of activated cells and number of Ca^2+^ microdomains within the first 15s after bead stimulation. Data are presented similarly to **Figure 2** (means ± SEM; WT, n = 24 cells; C2, n = 33 cells). Statistical testing by two-tailed Mann-Whitney-U-test, *p<0.05. **(C)** Number of signals as a function of time for WT and C2 Jurkat T cells, averaged across the 24 WT and the 33 C2 clone data. Statistical testing by two-tailed Mann-Whitney-U-test, ****p<0.0001. **(D, E)** Dartboard projection plots for the WT (top) and C2 clone cell sets [bottom; same cells as used for **(B)** and **(D)**], showing the location and frequency Ca^2+^ microdomains within the first 5 seconds after bead contact **(D)** and at a refined time scale [**(E)**; moving average computation with a 1s interval].

The data processing pipeline used to analyze the Jurkat T cell data set was identical to the workflow described for the primary T cells in section 1. The analysis results are shown in **Figure 3**. **Figure 3A** illustrates the successful application of the shape normalization to the ratio images for both WT and C2 cells. The distribution of the visible Ca^2+^ signals and signal patterns inside the cells was preserved after normalization, and the microdomain size and morphology differed in accordance with the deformation of the cells and their local deviation from an optimal circle before shape normalization.

The global Ca^2+^ microdomain statistics shown in **Figures 3B, C** agree with the corresponding results in (7): The average number of signals after bead contact was significantly higher in the WT cells compared to the C2 cells. In addition, for the WT cells, a peak of the Ca^2+^ microdomain number can be seen at around 4 to 5 s after bead contact (**Figure 3C**). Different from the data shown by Roggenkamp et al., the present analysis was based on the shape-normalized ratio images, supporting that the shape normalization does not negatively affect Ca^2+^ microdomain representation and detection.

However, the shape normalization enabled dartboard projections and, therefore, the analysis of local spatiotemporal Ca^2+^ signal distributions in the considered cell groups. In **Figure 3D**, the detected Ca^2+^ microdomains were aggregated over the first 5 seconds after bead contact. For the knockout cells, the dartboard showed that the detected signals were not located close to the bead contact area. For the WT cells, two main compartments were visible: Ca^2+^ microdomains in the dartboard regions close to the bead contact and microdomains in and near the dartboard bullseye, that is, in the cell centers. Increasing the temporal resolution of the dartboard projections, as shown in **Figure 3E**, finally illustrated for the WT cells that the microdomains close to the bead contact were primarily identified directly after bead contact, while the microdomains in the cell centers correlated to the described peak around 4-5 s in **Figure 3C**. For the knockout cells, no such observations were made.

### 3.3 Human NK cells: time-dependent study of signal

The examples in sections 3.1 and 3.2 focused on Ca^2+^ microdomain formation in T cells. However, Ca^2+^ microdomain formation is also relevant in other cell types and contexts, and the application of DARTS is not restricted to T cells. In this third application example, DARTS was applied to Ca^2+^ microdomain analysis in NK cells, a cell type, for which such localized Ca^2+^ signals have not been visualized and comprehensively analyzed so far.

#### 3.3.1 Data set description

KHYG-1 cells, a human NK cell line, were loaded with Fluo4-AM (10 µM) and Fura Red-AM (20 µM) for ratiometric single-cell Ca^2+^ imaging. KHYG-1 cells were stimulated with protein G beads (Merck Millipore), coated with a combination of antibodies (anti-NKp46 and anti-2B4) for a localized activation. The imaging set-up was identical to the setup described in section 3.1.

#### 3.3.2 Illustration of DARTS application

**Figure 4:**
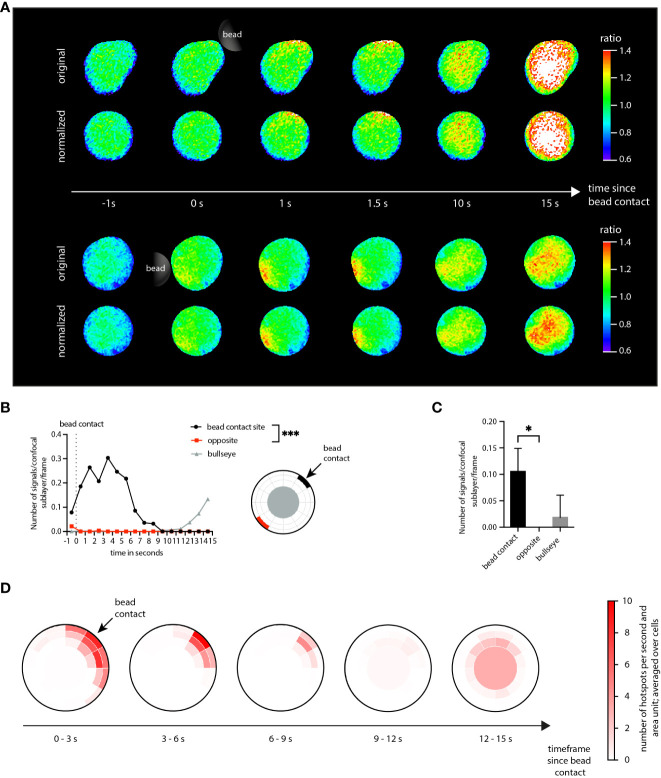
Application of DARTS to KHYG-1 cells. **(A)** Ca^2+^ signals in two exemplary KHYG-1 cells within the first seconds after stimulatory bead contact (top: original cell representation; bottom: shape-normalized cell representation). **(B)** Number of Ca^2+^ microdomains as a function of time for a dartboard area near the bead contact (black), opposite the bead contact (red), and the cell center (dartboard bullseye), averaged across n=7 cells. Statistical testing by two-tailed Mann-Whitney-U-test, ***p<0.001. **(C)** The corresponding average number of selected Ca^2+^ microdomains. Statistical testing by two-tailed Mann-Whitney-U-test, *p<0.05. **(D)** Dartboard projection plots, averaged over the n=7 cells and different time frames.

The analysis workflow applied to the KHYG-1 cells was the same as the one used for the Jurkat T cells. As visible for the NK cell shown in the top row of **Figure 4**, aggregation of localized Ca^2+^ signals in a NK cell set for subsequent dartboard projections required shape normalization for the cells. The central scientific questions in the present analysis scenario were: Is it possible to visualize initial Ca^2+^ microdomains in NK cells stimulated with antibody-coated beads? If so, can the Ca^2+^ microdomains be spatially and temporally associated with the stimulating bead contact?

To answer the first question, global microdomain statistics would have been sufficient. However, the dartboard projection provides the means to simultaneously answer both questions. Here, a microdomain analysis was performed in three dartboard areas: (1) close to the bead contact site, (2) for the bullseye, and (3) for the opposite side of the bead contact site (**Figure 4B**). The analysis results summarized in **Figure 4C** show that not only Ca^2+^ microdomains were detected in the NK cells, but that these were mainly located close to the bead contact. **Figure 4B** further illustrates that the detected bullseye microdomains arose later, approximately 10 s after bead contact. **Figure 4D** provides the corresponding comprehensive dartboard projections, further strengthening the observations shown in **Figures 4C, B**.

Thus, both scientific questions could be answered with yes. Here, the dartboard projections could be used to define inner-cell control areas to demonstrate that the initial Ca^2+^ dynamics can be directly associated with the bead contact.

## 4 Discussion

In the present work, we introduced DARTS, an open-source Python pipeline to process fluorescence microscopy live cell imaging data with the specific focus on the analysis of Ca^2+^ microdomains. DARTS integrates state-of-the-art deep learning methods for cell detection and segmentation and provides cell shape normalization and a so-called dartboard projection to facilitate intuitive visualization and comparison of Ca^2+^ microdomain dynamics within and across different cell data sets.

The application of DARTS was demonstrated by means of three examples. The examples illustrate that the dartboard analysis and visualization enables aggregation of locally resolved information that helps to compare and understand the spatio-temporal Ca^2+^ dynamics at different time points and intervals post-bead stimulation for different cell groups. This, in turn, is essential for a better understanding of the underlying cellular processes. Residual quantitative differences in, for instance, the number of hotspots compared to the corresponding quantitative results in (3, 7) are due to implementation differences of post-processing modules, such as an altered order of the deconvolution and bleaching correction and changes of the deconvolution implementation. Moreover, the shape normalization is expected to change the results of the hotspot detection slightly, depending on the deviation from a perfect circle. However, these low-level differences were negligible for the application examples, demonstrating the robustness of the implemented methods.

As shown in the application examples, the primary use case of DARTS is to analyze Ca^2+^ microdomain dynamics for groups of bead-activated cells. However, it can also be used to define intra-cellular control areas, for instance to answer the question if observed Ca^2+^ dynamics are more pronounced near the bead contact than farther away (cf. application example 3). Thus, the DARTS analysis options provide a more holistic view than the related usual global measures.

Despite its current application focus, different DARTS modules and analysis options are expected to be of interest for different experiments and analysis scenarios. Potential use cases are, for instance, a microdomain analysis for different cell activation approaches like microinjection. General methodical aspects of DARTS like the provided deconvolution approaches and the shape normalization can also be applied to live cell imaging data acquired in an entirely different experimental setting. The application is not limited to immune cells alone, other cell types such as neurons are also expected to work with the framework. For the shape normalization, however, a minimal convexity/circularity is required. Similarly, the trigger of the Ca^2+^ release does not need to be a bead contact. The postprocessing and analysis steps can also be used without bead contact definition.

**Figure 5:**
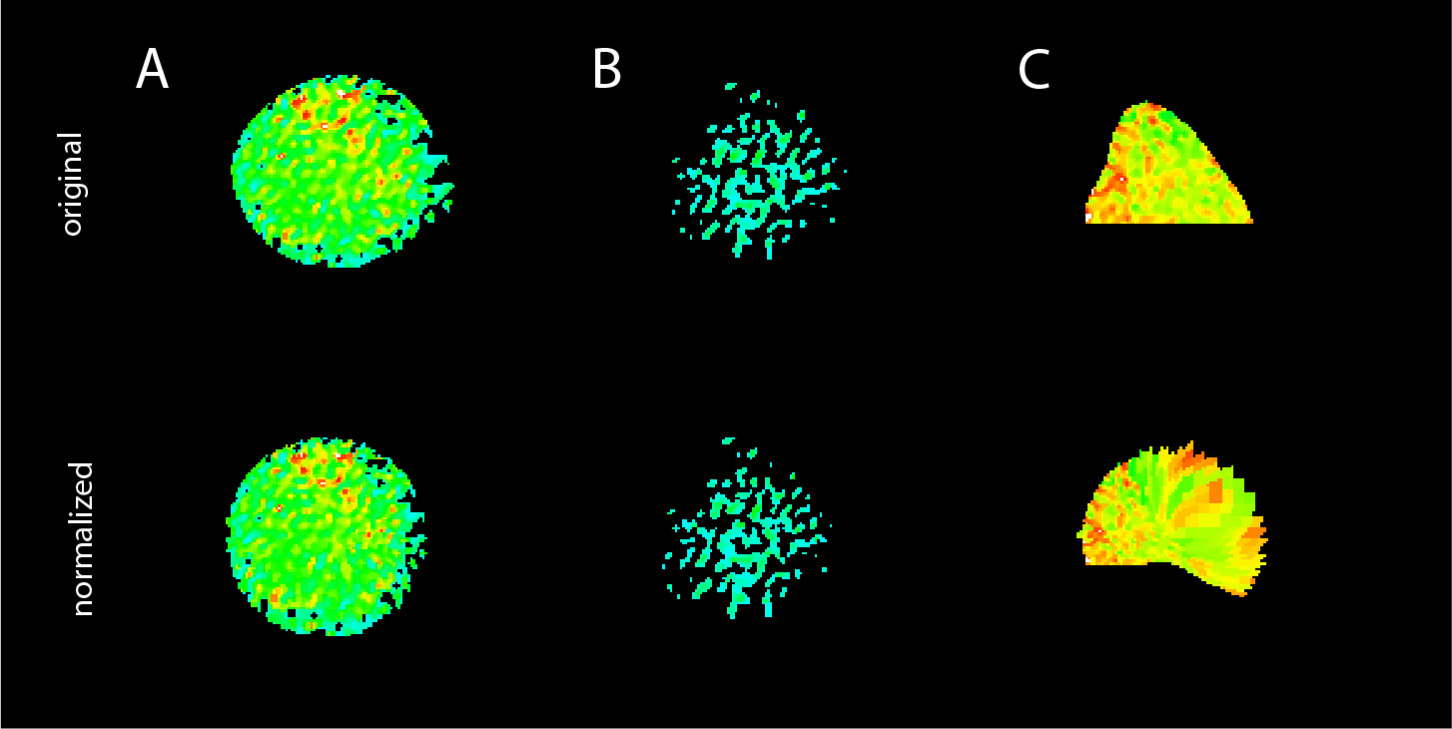
Limitations of the DARTS pipeline. Examples of failed processing: **(A)** frayed cell edges and **(B)** “vanishing” cell, both due to erroneous threshold-based background subtraction; **(C)** failed cell shape normalization for a cell that was not entirely within the region of interest to be processed.

DARTS will be continuously extended. Current developments comprise the autodetection of bead contacts, adapting state-of-the-art machine learning-based methods like in (25), as well as integration of further deconvolution and denoising approaches. Moreover, options for autodetection of rare shape normalization failures (see **Figure 5** for examples) will also be incorporated. In each case: Since DARTS is provided as an open-source solution, the next implementation steps and extensions will also depend on and be aligned with the needs of the community. Feedback and suggestions to optimize the DARTS pipeline and its modules are very much welcome.

## Data availability statement

The raw data supporting the conclusions of this article will be made available by the authors, without undue reservation.

## Author contributions

LW: Conceptualization, Formal analysis, Software, Validation, Visualization, Writing – original draft, Writing – review & editing. DK: Formal analysis, Investigation, Software, Validation, Visualization, Writing – original draft, Writing – review & editing. HH: Formal analysis, Software, Writing – review & editing. FF: Software, Writing – review & editing. FG: Formal analysis, Investigation, Writing – review & editing. FM: Formal analysis, Investigation, Writing – review & editing. MA: Funding acquisition, Writing – review & editing. AG: Funding acquisition, Writing – review & editing. BD: Data curation, Formal analysis, Funding acquisition, Project administration, Supervision, Validation, Writing – review & editing. RW: Conceptualization, Funding acquisition, Project administration, Supervision, Validation, Writing – review & editing.
